# Supplementary material for: Genome-wide association analyses of chronotype in 697,828 individuals provides insights into circadian rhythms
Source: Nat Commun. 2019 Jan 29;10:343. doi: 10.1038/s41467-018-08259-7 (PMC6351539; doi:10.1038/s41467-018-08259-7)
Supplement: Supplementary file 1 — Supplementary Information [file 41467_2018_8259_MOESM1_ESM.pdf]

Genome-wide association analyses of chronotype in 697,828 individuals provides insights into circadian rhythms

Jones, Lane, Wood et al

Supplementary Methods

Identifying a significance threshold using permutation testing

To identify a reasonable GWAS P-value threshold, we performed GWAS of 20 randomly permuted versions of our chronotype phenotype. The permuted phenotypes were created by randomly shuffling the raw chronotype values amongst the 451,454 white European participants with genetic data. We performed GWAS using BOLT-LMM, adjusting for for age, sex, study centre (categorical) and “release” (see “Genome-wide association analysis” subsection of the **Methods**), as with the original phenotype. From these 20 GWAS, a total of six variants reached genome-wide significance. The table below gives the lowest *P* and corresponding -log10(*P*) per permutation.

**Permutation**	P_{min}	-log10(P_{min})
1	4.80E-09	8.318758763
2	1.40E-07	6.853871964
3	8.10E-07	6.091514981
4	4.70E-08	7.327902142
5	1.50E-07	6.823908741
6	1.60E-08	7.795880017
7	5.50E-08	7.259637311
8	6.90E-07	6.161150909
9	2.00E-07	6.698970004
10	5.80E-08	7.236572006
11	2.10E-07	6.677780705
12	5.30E-08	7.27572413
13	7.30E-08	7.13667714
14	2.50E-08	7.602059991
15	4.50E-07	6.346787486
16	2.00E-07	6.698970004
17	3.20E-07	6.494850022
18	2.50E-08	7.602059991
19	1.60E-06	5.795880017
20	4.20E-07	6.37675071

To identify a suitable threshold to ensure our type I error rate (alpha) remains at 0.05 (as assumed by the threshold of $5x10^{-8}$ for a million independent tests), we can take an average using either the median or mean of the -log10(P_{min}). We then transform this average back to a *P*-value, and multiply the result by our alpha of 0.05, using the result as a significance threshold for our GWAS. Taking the median gives median(-log10(P_{min})) = 6.84. Converting this to a *P*-value gives $1.45x10^{-7}$ and multiplying by an alpha of 0.05 gives a *P*-value threshold of $7x10^{-9}$. Using the mean instead gives mean(log10(P_{min})) = 6.93, giving a *P*-value of $1.2x10^{-7}$ and so a threshold of $6x10^{-9}$.

Sensitivity analysis details

Shift or night shift work

Shift and night shift work were assessed using two questionnaire variables recorded at baseline. If individuals had answered anything other than “Never/rarely” for either field 826 (“Job involves shift work”) or field 3426 (“Job involves night shift work”), they were considered to be shift or night shift workers at baseline and were excluded.

Self-report mental health and sleep disorders

Individuals were excluded if they answered yes to field 2090 (“Seen doctor (GP) for nerves, anxiety, tension or depression”) at baseline or had reported “depression”, “schizophrenia”, “mania/bipolar disorder/manic depression”, “sleep apnoea” or “insomnia” at the baseline verbal interview. This data is stored in field 20002 (“Non-cancer illness code, self-reported”).

Hospital episode statistics (HES) mental health and sleep disorder diagnoses

We excluded individuals if they had any one of the following diagnoses codes (and corresponding subclassifications) in fields 41202 (primary diagnoses) or 41204 (secondary diagnoses):

- Major depressive disorder (MDD) – ICD10 codes F32 and F33
- Schizophrenia – F20 to F29
- Bipolar disorder – F30 and F31
- Anxiety disorders – F40 to F43
- Mood disorders – F30 to F39
- Sleep disorders – F51 (nonorganic) and G47

Relevant self-report medications

Individuals were excluded if they reported any of the following medications at baseline (field 20003):

Sleep medications: oxazepam, meprobamate, medazepam, bromazepam, lorazepam, clobazam, chlormezanone, temazepam, nitrazepam, lormetazepam, diazepam, zopiclone, triclofos, methyprylone, prazepam, triazolam, ketazolam, dichloralphenazone, clomethiazole, zaleplon, butobarbital, diphenhydramine product, nytol, sonata

Antidepressants: amitriptyline, citalopram, fluoxetine, sertraline, venlafaxine, dosulepin, paroxetine, mirtazapine, escitalopram, trazodone, prozac, seroxat, cipralex, duloxetine, lofepramine, clomipramine, nortriptyline, imipramine, dothiepin, cipramil, amitriptyline, prothiaden, trimipramine, lustral, reboxetine, zispin, cymbalta, anafranil, doxepin, moclobemide, phenelzine, fluvoxamine, yentreve, triptafen, surmontil, tranylcypromine, allegron, edronax, molipaxin, mianserin, nardil, faverin, nefazodone, amitriptyline+chlordiazepoxide, isocarboxazid, manerix, maoi, sinequan, tranylcypromine+trifluoperazine, ludiomil, norval, tryptizol, fluphenazine hydrochloride+nortriptyline.

Antipsychotics: prochlorperazine, olanzapine, quetiapine, risperidone, chlorpromazine, trifluoperazine, amisulpride, sulpiride, seroquel, haloperidol, aripiprazole, stelazine, depixol, flupentixol, clozapine, promazine, risperdal, modecate, fluanxol, flupenthixol, zyprexa,

zuclopenthixol, clopixol, largactil, abilify, fluphenazine, haldol, serenace, clozaril, cpz, perphenazine, levomepromazine, pericyazine, dolmatil, fentazin, fluphenazine, benperidol, pimozide, zaponex, denzapine, neulactil, thioridazine, dozic, fluspirilene, panadeine, sertindole.

Anxiolytics: zopiclone, diazepam, temazepam, zolpidem, nitrazepam, lorazepam, hydroxyzine, zimovane, phenergan, promethazine, buspirone, atarax, oxazepam, loprazolam, chlordiazepoxide, lormetazepam, ucerax, stilnoct, diazepam, buspar, alprazolam, librium, xanax, meprate, dalmane, clomethiazole, meprobamate, welldorm, amitriptyline+chlordiazepoxide, flurazepam, heminevrin, medazepam, neulactil, sinequan, almazine, atensine, carisoma, chloractil, chloral, dichloralphenazone, dormonoct, methyprylone, mogadon, rohypnol, tryptizol

Supplementary Tables

Supplementary Table 1. Basic summary statistics and observational associations for the raw sleep and activity measures. Units for L5, M10, sleep duration (mean and SD), sleep midpoint and diurnal inactivity are in hours. Sleep efficiency is a ratio and number of sleep episodes is a count.

	Overall					Morning Person			Evening Person			
**Measure (beta unit)**	**Mean**	**SD**	**Min**	**Max**	**N***	**Mean**	**SD**	**N***	**Mean**	**SD**	**N***	**P****
L5 timing (hrs from previous midnight)	27.32	1.07	12.29	35.35	85830	27.07	0.98	41298	27.72	1.11	23820	<1E-300
M10 timing (hrs from previous midnight)	13.70	1.21	0.26	23.44	85723	13.43	1.12	41256	14.13	1.26	23791	<1E-300
Sleep midpoint (hrs from previous midnight)	26.99	0.91	16.25	31.98	85502	26.81	0.84	41307	27.26	0.98	23826	<1E-300
Sleep duration mean (hrs)	7.30	0.86	3.00	11.87	85502	7.31	0.86	41307	7.26	0.89	23826	2.0E-06
Sleep duration SD (hrs)	0.93	0.57	0.00	7.26	85068	0.92	0.56	41113	0.97	0.59	23699	9.0E-18
Sleep efficiency (%)	0.76	0.07	0.29	1.07	85502	76.20	7.17	41307	75.98	7.46	23826	2.0E-04
Number of sleep episodes (N)	17.25	3.59	5.14	29.86	85502	17.21	3.57	41307	17.25	3.65	23826	9.0E-02
Diurnal inactivity duration (hrs)	0.97	0.68	0.00	9.21	85502	0.943	0.65	41307	1.01	0.72	23826	2.0E-38

*Unrelated white Europeans only

**Adjusted for age at study and sex

Supplementary Table 2. Chronotype genetic risk score associations and directional consistency with the raw actigraphy traits. Each GRS was tested using the regression model: TRAIT ~ GRS + age + genotype release + season + number of measurements. GRS betas are reported per unit standard deviation increase in GRS with a higher GRS indicating greater odds of morningness. Consistency was tested as morningness increasing/trait decreasing. Meta-analysis GRS and directional consistency considered only the 292 lead variants identified in the meta-analysis (and available in UK Biobank) that excluded the activity monitor participants, in order to avoid inflation of results.

	Meta-analysis GRS			23andMe GRS			Meta-analysis Directional Consistency		23andMe Directional Consistency	
**Measure (beta unit)**	**Beta**	**SE**	**P**	**Beta**	**SE**	**P**	**N_{con}/N_{total}**	**P**	**N_{con}/N_{total}**	**P**
L5 timing (hrs from previous midnight)	-0.1046	0.0036	1.0E-182	-0.0775	0.0036	2.0E-100	262/292	2.1E-47	100/109	1.4E-20
M10 timing (hrs from previous midnight)	-0.0996	0.0041	7.0E-130	-0.0724	0.0041	4.9E-69	260/292	1.5E-45	96/109	8.3E-17
Sleep midpoint (hrs from previous midnight)	-0.0747	0.0031	4.0E-128	-0.0559	0.0031	3.3E-72	258/292	8.8E-44	100/109	1.4E-20
Sleep duration mean (hrs)	-0.0011	0.0029	7.1E-01	0.0044	0.0029	1.3E-01	150/292	6.8E-01	50/109	4.4E-01
Sleep duration s.d. (hrs)	-0.0039	0.0019	3.7E-02	-0.0035	0.0019	6.6E-02	166/292	2.2E-02	61/109	2.5E-01
Sleep efficiency (%)	-0.0003	0.0002	2.0E-01	0.0002	0.0002	4.5E-01	118/292	1.2E-03	34/109	1.1E-04
Number of sleep episodes (N)	0.0122	0.0122	3.1E-01	-0.0123	0.0122	3.1E-01	148/292	8.6E-01	54/109	1.0E+00
Diurnal inactivity duration (hrs)	-0.0045	0.0023	4.8E-02	-0.0045	0.0023	4.9E-02	160/292	1.1E-01	63/109	1.3E-01

Supplementary Table 3. GTEx tissue enrichment results from FUMA (MAGMA). Tissues highlighted green have Bonferroni corrected P values < 0.05.

GTEx Tissue	Beta	Beta SD	SE	P	P Bonf
Brain_Cerebellar_Hemisphere	0.112	0.284	0.0117	9.82E-22	5.2E-20
Brain_Cerebellum	0.112	0.285	0.0122	3.21E-20	1.7E-18
Brain_Frontal_Cortex_BA9	0.103	0.254	0.0134	7.86E-15	4.16E-13
Brain_Cortex	0.103	0.253	0.0139	6.52E-14	3.46E-12
Brain_Anterior_cingulate_cortex_BA24	0.104	0.248	0.0143	1.63E-13	8.62E-12
Brain_Nucleus_accumbens_basal_ganglia	0.104	0.246	0.015	2.69E-12	1.42E-10
Brain_Hypothalamus	0.104	0.247	0.0164	1.57E-10	8.33E-09
Brain_Caudate_basal_ganglia	0.0997	0.236	0.016	2.17E-10	1.15E-08
Brain_Putamen_basal_ganglia	0.0968	0.223	0.016	7.21E-10	3.82E-08
Brain_Amygdala	0.0955	0.221	0.016	1.18E-09	6.25E-08
Brain_Hippocampus	0.094	0.217	0.0161	2.77E-09	1.47E-07
Pituitary	0.0783	0.198	0.0185	1.15E-05	0.000608
Brain_Substantia_nigra	0.0729	0.169	0.0182	3.26E-05	0.001726
Brain_Spinal_cord_cervical_c.1	0.0524	0.12	0.018	0.0018383	0.09743
Cells_EBV.transformed_lymphocytes	0.0131	0.0346	0.00941	0.081659	1
Muscle_Skeletal	-0.00392	-0.00893	0.0132	0.61692	1
Testis	-0.00319	-0.00846	0.0103	0.62154	1
Ovary	-0.00754	-0.0194	0.0218	0.6354	1
Artery_Tibial	-0.0286	-0.0716	0.0232	0.89092	1
Colon_Sigmoid	-0.0383	-0.096	0.0306	0.89453	1
Adrenal_Gland	-0.0294	-0.0751	0.0216	0.91374	1
Whole_Blood	-0.0149	-0.0319	0.0103	0.92583	1
Cells_Transformed_fibroblasts	-0.0229	-0.0583	0.015	0.93641	1
Uterus	-0.0439	-0.113	0.0282	0.9401	1
Esophagus_Gastroesophageal_Junction	-0.0677	-0.17	0.0326	0.98115	1
Esophagus_Muscularis	-0.0645	-0.161	0.0305	0.98283	1
Nerve_Tibial	-0.0558	-0.146	0.0259	0.98424	1
Cervix_Endocervix	-0.0706	-0.182	0.0313	0.98788	1
Spleen	-0.0351	-0.0913	0.0148	0.99096	1
Pancreas	-0.0429	-0.0934	0.0175	0.9929	1
Thyroid	-0.0604	-0.157	0.0239	0.99433	1
Prostate	-0.0895	-0.228	0.0309	0.99814	1
Cervix_Ectocervix	-0.105	-0.262	0.0352	0.99858	1
Heart_Left_Ventricle	-0.0544	-0.123	0.0182	0.99863	1
Small_Intestine_Terminal_Ileum	-0.054	-0.135	0.0179	0.99868	1
Artery_Aorta	-0.0736	-0.184	0.024	0.99892	1
Colon_Transverse	-0.0776	-0.191	0.0253	0.99894	1
Heart_Atrial_Appendage	-0.0652	-0.154	0.0211	0.99902	1
Stomach	-0.0934	-0.224	0.0294	0.99926	1
Liver	-0.0377	-0.0862	0.0118	0.9993	1
Bladder	-0.106	-0.271	0.0311	0.99969	1
Artery_Coronary	-0.106	-0.266	0.0301	0.99978	1

Fallopian_Tube	-0.122	-0.313	0.0324	0.99992	1
Skin_Not_Sun_Exposed_Suprapubic	-0.0631	-0.159	0.0166	0.99993	1
Skin_Sun_Exposed_Lower_leg	-0.0633	-0.16	0.0165	0.99994	1
Adipose_Subcutaneous	-0.102	-0.262	0.0257	0.99996	1
Breast_Mammary_Tissue	-0.134	-0.342	0.0335	0.99997	1
Minor_Salivary_Gland	-0.0928	-0.226	0.0221	0.99999	1
Vagina	-0.125	-0.313	0.0288	0.99999	1
Adipose_Visceral_Omentum	-0.131	-0.331	0.0269	1	1
Esophagus_Mucosa	-0.0701	-0.173	0.0157	1	1
Kidney_Cortex	-0.0937	-0.229	0.0208	1	1
Lung	-0.102	-0.261	0.0214	1	1

Supplementary Table 4. Enrichment of SCN-enriched and fluctuating genes at Chronotype loci and loci associated with six other phenotypes. Associated variants were mapped to genes using MAGMA. Lists of SCN-enriched and fluctuating expression genes were obtained from Supplementary Table 2 of Pembroke et al., 2015, eLife, https://doi.org/10.7554/eLife.10518 and Supplementary Table 1 of Brown et al., 2017, Nucleic Acids Res, https://dx.doi.org/10.1093/nar/gkx714. For each phenotype, only genes that were both mapped using MAGMA and tested in each study were included.

	SCN-enriched Genes					Fluctuating Genes				
**Phenotype**	**N enriched**	**N not enriched**	**N total**	**Proportion enriched**	**P**	**N fluctuating**	**N not fluctuating**	**N total**	**Proportion fluctuating**	**P**
**All Genes Tested**	7131	24553	31684	0.225	-	5017	24804	29821	0.168	-
**Chronotype**	225	579	804	0.280	3.00E-04	251	534	785	0.320	1.25E-28
**Schizophrenia**	30	73	103	0.291	1.24E-01	27	69	96	0.281	3.15E-03
**Crohn's Disease**	54	192	246	0.220	8.78E-01	62	178	240	0.258	2.07E-04
**BMI**	84	257	341	0.246	3.62E-01	88	240	328	0.268	1.54E-06
**Birthweight**	438	1418	1856	0.236	2.78E-01	482	1301	1783	0.270	2.29E-28
**Menopause Age**	153	556	709	0.216	5.85E-01	188	484	672	0.280	2.99E-14
**Height**	1452	4725	6177	0.235	8.71E-02	1673	4226	5899	0.284	1.17E-95

Supplementary Figures

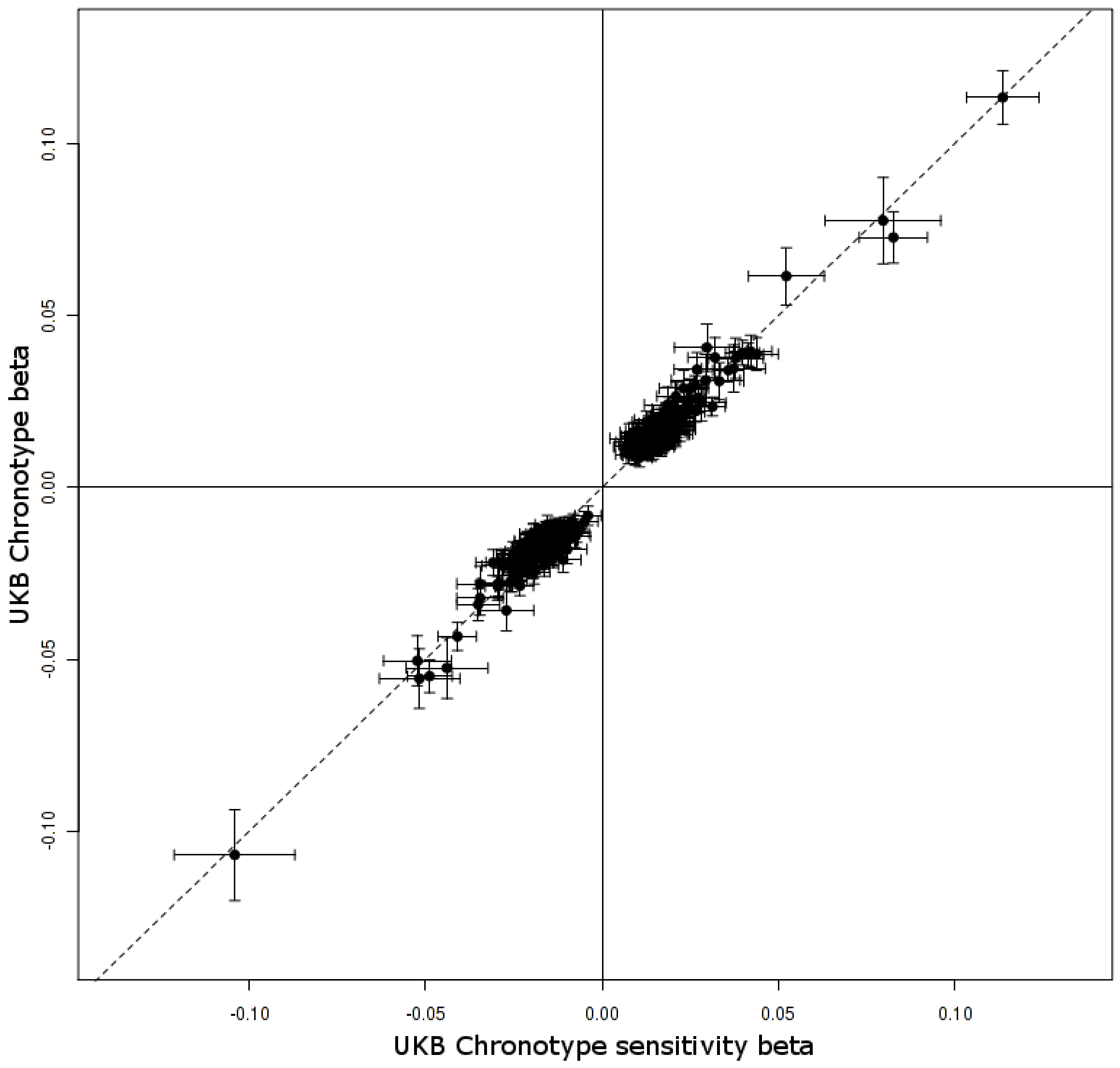

Supplementary Figure 1. Chronotype lead variant effect sizes vs. sensitivity GWAS effect sizes. The plot shows UK Biobank chronotype GWAS effect sizes for all 341 (of 351) meta-analysis lead variants present in UK Biobank, against their effect sizes in the UK Biobank chronotype sensitivity GWAS. The dashed line indicates identical effect in both analyses. Error bars represent standard errors of effect size estimates.

a)

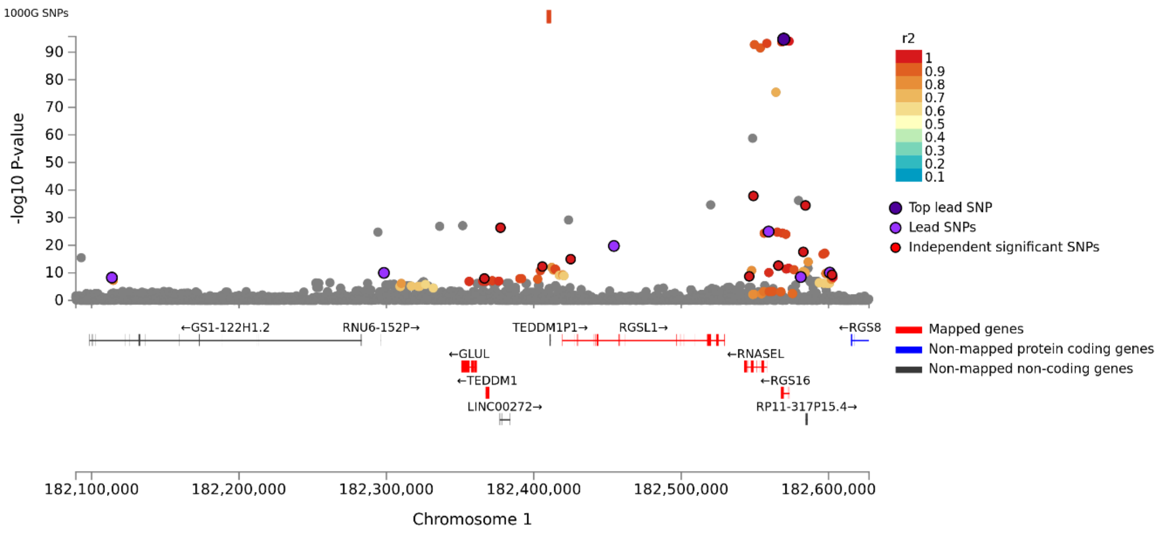
1000G SNPs
-log10 P-value
0
10
20
30
40
50
60
70
80
90
r2
1
0.9
0.8
0.7
0.6
0.5
0.4
0.3
0.2
0.1
Top lead SNP
Lead SNPs
Independent significant SNPs
←GS1-122H1.2
RNU6-152P→
TEDDM1P1→
RGSL1→
←RGS8
←GLUL
←RNASEL
←TEDDM1
←RGS16
LINC00272→
RP11-317P15.4→
Mapped genes
Non-mapped protein coding genes
Non-mapped non-coding genes
182,100,000
182,200,000
182,300,000
182,400,000
182,500,000
182,600,000
Chromosome 1

b)

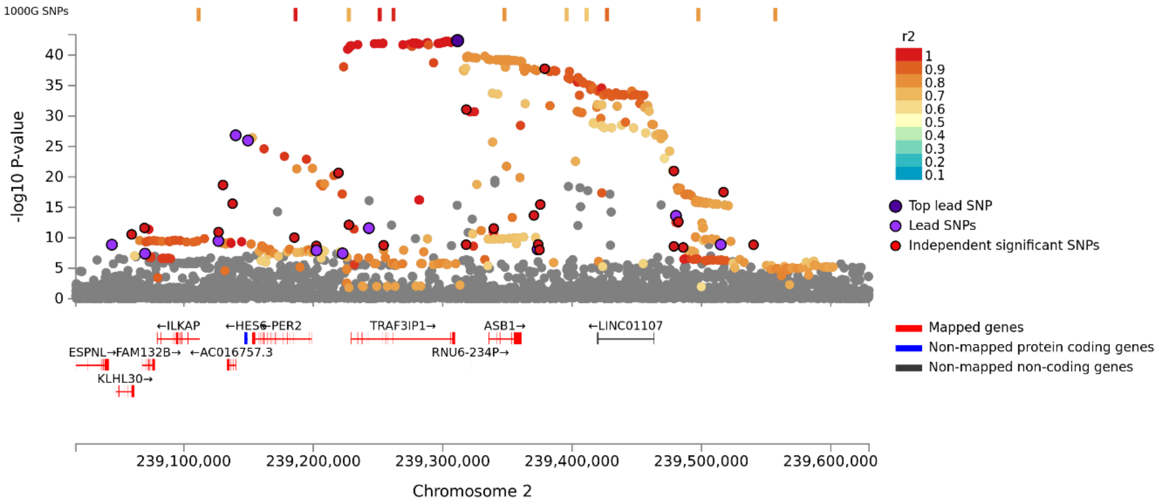
1000G SNPs
-log10 P-value
0
5
10
15
20
25
30
35
40
r2
1
0.9
0.8
0.7
0.6
0.5
0.4
0.3
0.2
0.1
Top lead SNP
Lead SNPs
Independent significant SNPs
←ILKAP
←HES6
PER2
TRAF3IP1→
ASB1→
←LINC01107
ESPNL→
FAM132B→
←AC016757.3
RNU6-234P→
KLHL30→
Mapped genes
Non-mapped protein coding genes
Non-mapped non-coding genes
239,100,000
239,200,000
239,300,000
239,400,000
239,500,000
239,600,000
Chromosome 2

c)

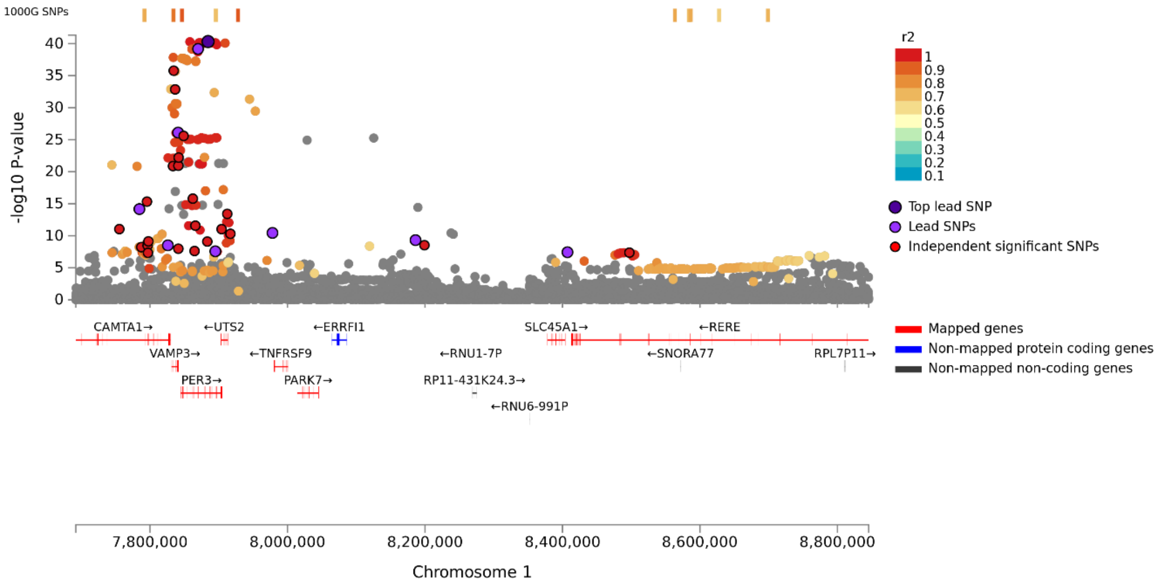
1000G SNPs
-log10 P-value
40
35
30
25
20
15
10
5
0
CAMTA1→
←UTS2
←ERRFI1
SLC45A1→
←RERE
VAMP3→
←TNFRSF9
←RNU1-7P
←SNORA77
RPL7P11→
PER3→
PARK7→
RP11-431K24.3→
←RNU6-991P
7,800,000
8,000,000
8,200,000
8,400,000
8,600,000
8,800,000
Chromosome 1
r2
1
0.9
0.8
0.7
0.6
0.5
0.4
0.3
0.2
0.1
Top lead SNP
Lead SNPs
Independent significant SNPs
Mapped genes
Non-mapped protein coding genes
Non-mapped non-coding genes

d)

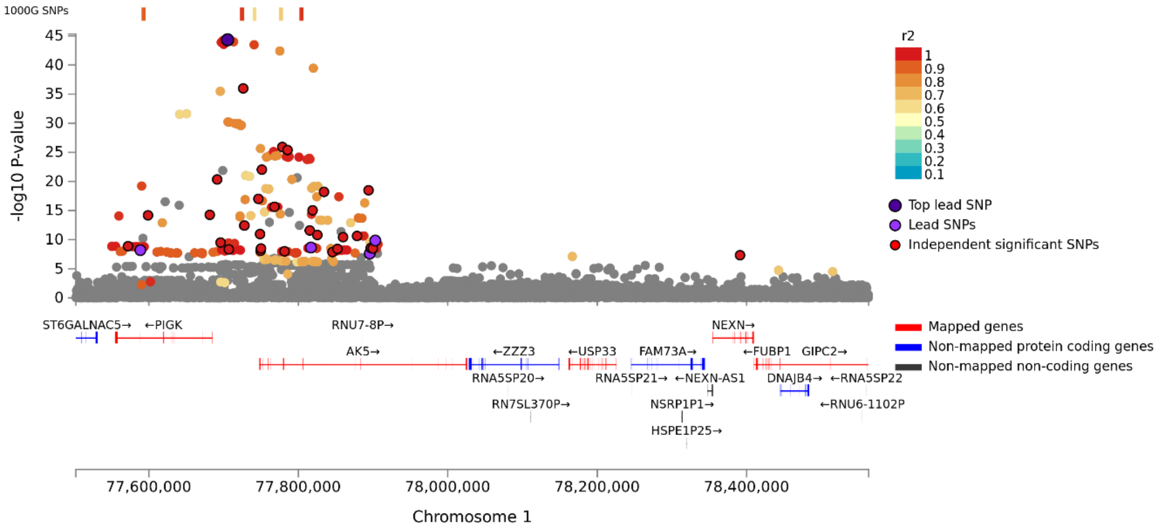
1000G SNPs
-log10 P-value
45
40
35
30
25
20
15
10
5
0
ST6GALNAC5→
←PIGK
RNU7-8P→
NEXN→
AK5→
←ZZZ3
←USP33
FAM73A→
←FUBP1
GIPC2→
RNA5SP20→
RNA5SP21→
←NEXN-AS1
DNAJB4→
←RNA5SP22
RN7SL370P→
NSRP1P1→
←RNU6-1102P
HSPE1P25→
77,600,000
77,800,000
78,000,000
78,200,000
78,400,000
Chromosome 1
r2
1
0.9
0.8
0.7
0.6
0.5
0.4
0.3
0.2
0.1
Top lead SNP
Lead SNPs
Independent significant SNPs
Mapped genes
Non-mapped protein coding genes
Non-mapped non-coding genes

e)

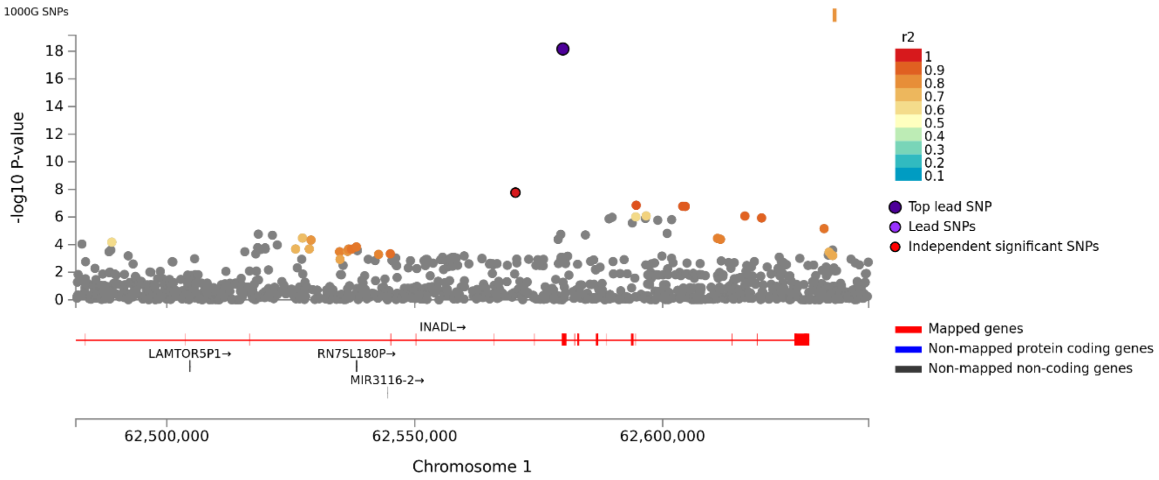

1000G SNPs
-log10 P-value
0
2
4
6
8
10
12
14
16
18
r2
1
0.9
0.8
0.7
0.6
0.5
0.4
0.3
0.2
0.1
Top lead SNP
Lead SNPs
Independent significant SNPs
INADL→
LAMTOR5P1→
RN7SL180P→
MIR3116-2→
Mapped genes
Non-mapped protein coding genes
Non-mapped non-coding genes
62,500,000
62,550,000
62,600,000
Chromosome 1

f)

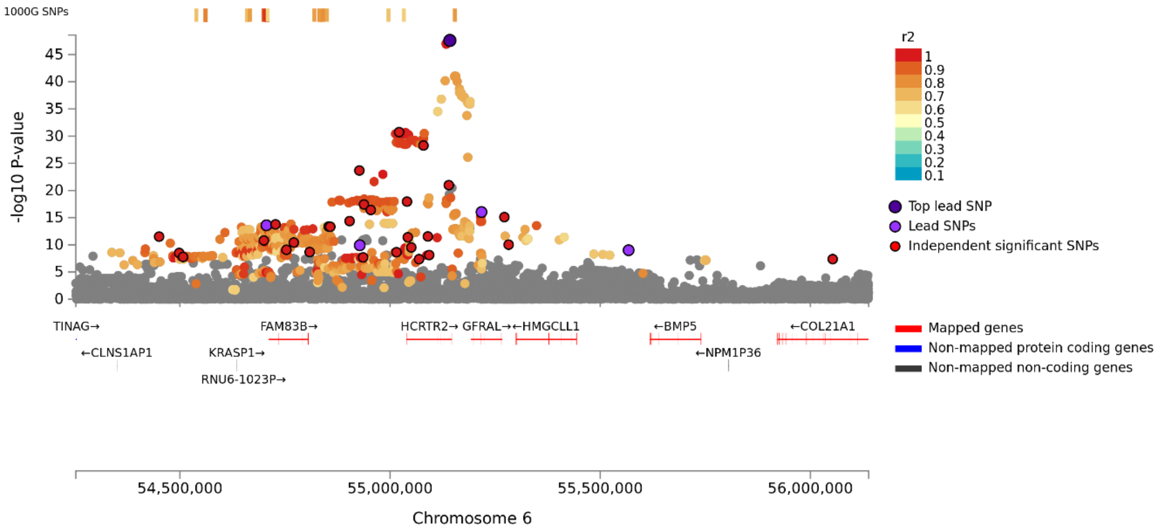

1000G SNPs
-log10 P-value
0
5
10
15
20
25
30
35
40
45
r2
1
0.9
0.8
0.7
0.6
0.5
0.4
0.3
0.2
0.1
Top lead SNP
Lead SNPs
Independent significant SNPs
TINAG→
FAM83B→
HCRTR2→
GFRAL→
←HMGCLL1
←BMP5
←COL21A1
←CLNS1AP1
KRASP1→
←NPM1P36
RNU6-1023P→
Mapped genes
Non-mapped protein coding genes
Non-mapped non-coding genes
54,500,000
55,000,000
55,500,000
56,000,000
Chromosome 6

g)

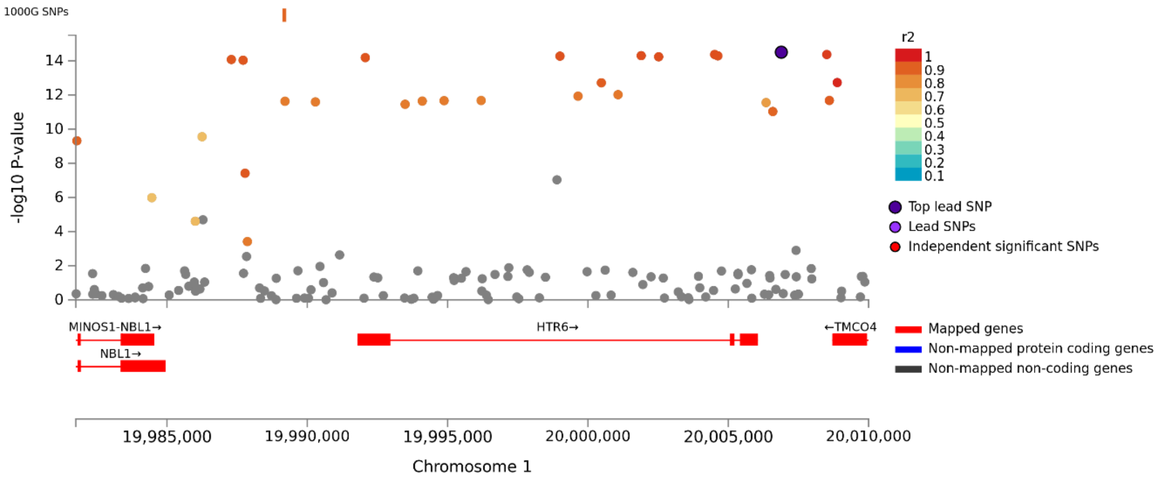
1000G SNPs
-log10 P-value
r2
Top lead SNP
Lead SNPs
Independent significant SNPs
MINOS1-NBL1→
NBL1→
HTR6→
←TMCO4
Mapped genes
Non-mapped protein coding genes
Non-mapped non-coding genes
19,985,000
19,990,000
19,995,000
20,000,000
20,005,000
20,010,000
Chromosome 1

h)

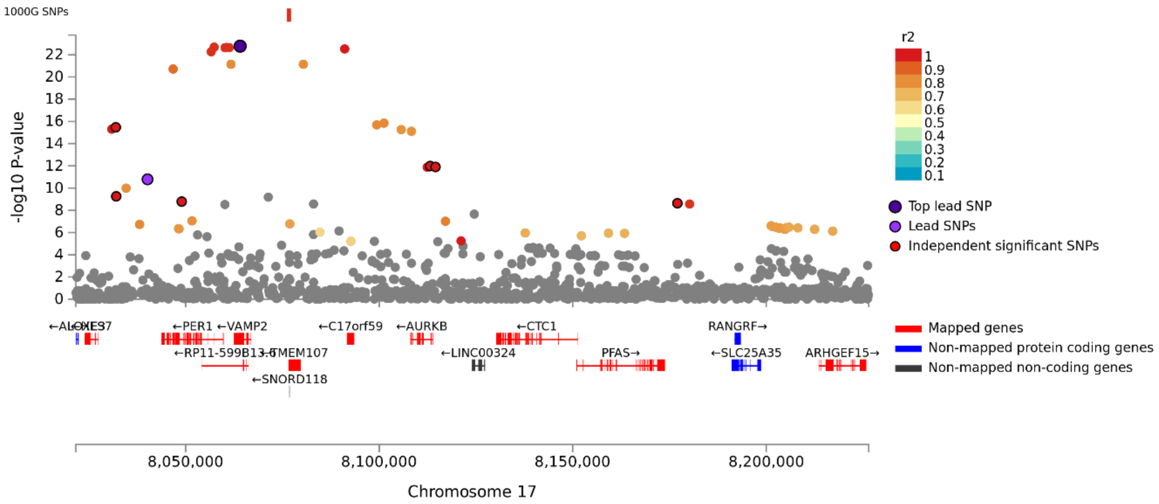
1000G SNPs
-log10 P-value
r2
Top lead SNP
Lead SNPs
Independent significant SNPs
←PER1 ←VAMP2
←C17orf59
←AURKB
←CTC1
RANGRF→
←RP11-599B13.6
←TMEM107
←LINC00324
PFAS→
←SLC25A35
ARHGEF15→
←SNORD118
Mapped genes
Non-mapped protein coding genes
Non-mapped non-coding genes
8,050,000
8,100,000
8,150,000
8,200,000
Chromosome 17

i)

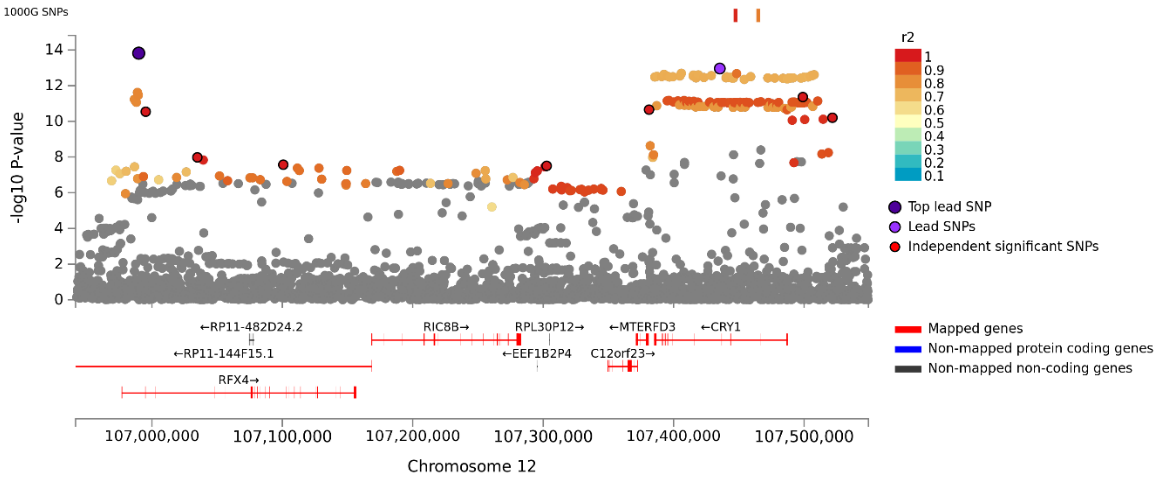

j)

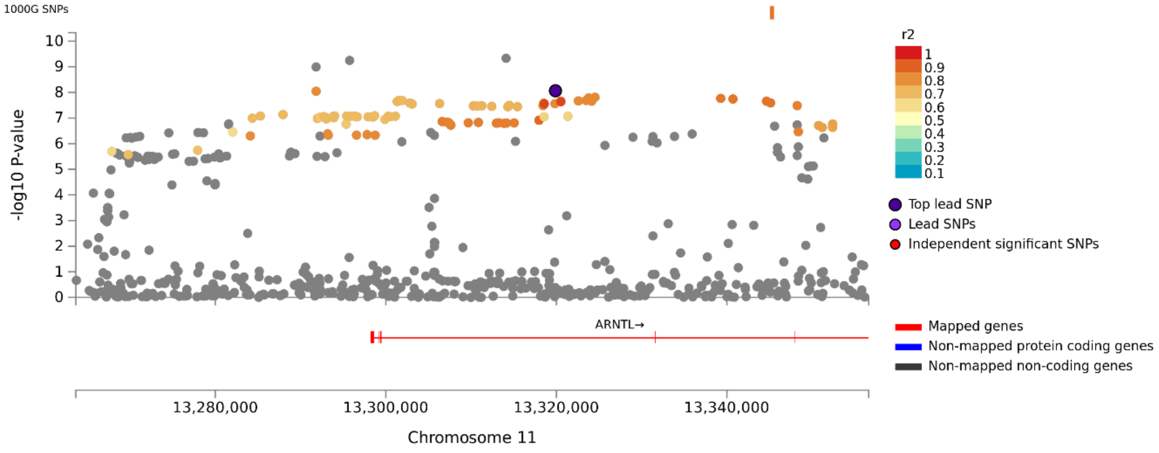

Supplementary Figure 2. FUMA regional plots of chronotype GWA results. Plots display the chronotype-associated variants at the a) RGS16, b) PER2/TRAF3IP1, c) PER3, d) PIGK/AK5, e) INADL, f) HCRTR2, g) HTR6, h) PER1, i) CRY1 and j) ARNTL loci. LD r^2 of each variant with the lead variant is indicated by the colour of the points, with mapped genes highlighted beneath in red.

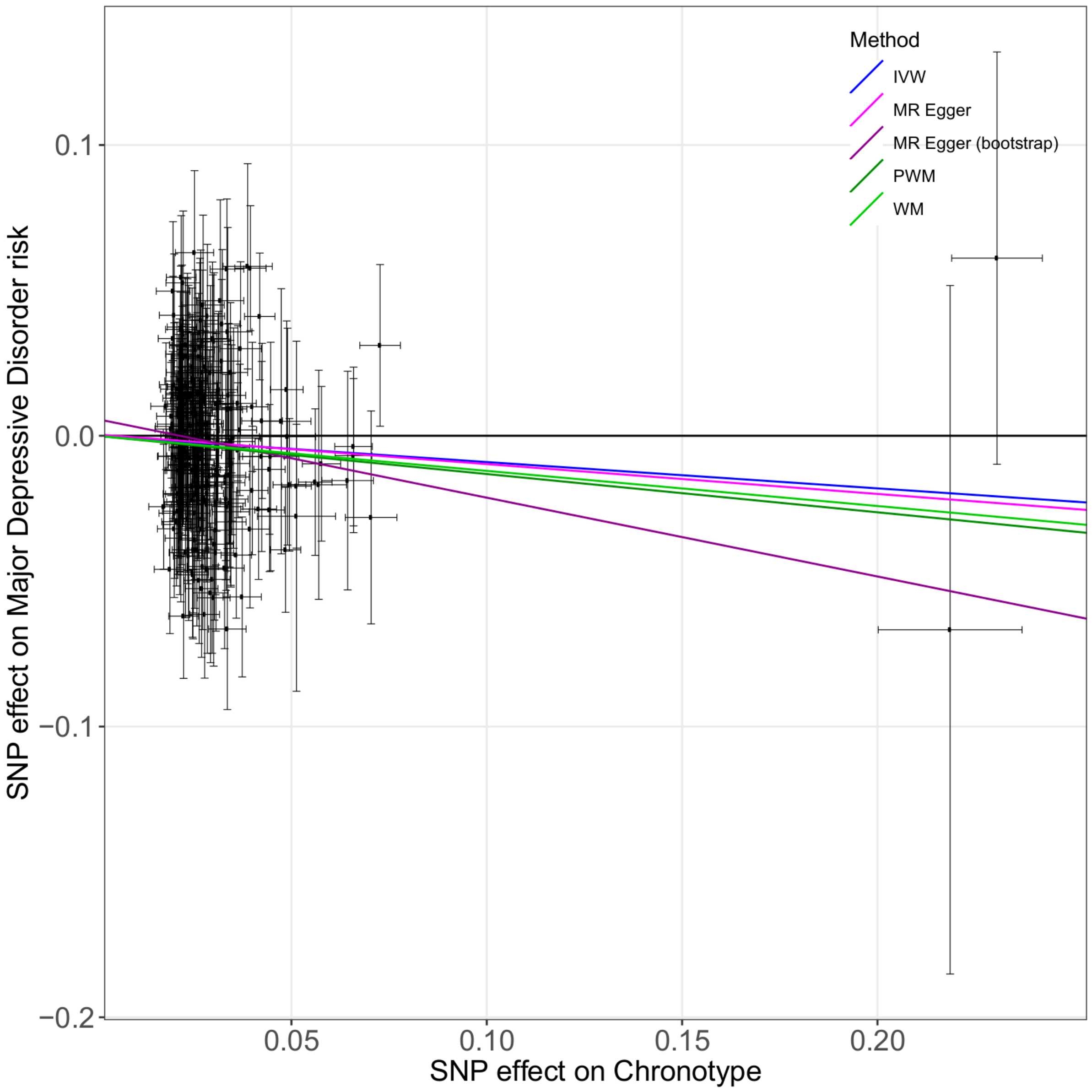

Supplementary Figure 3. MR scatter plot of major depressive disorder risk vs. chronotype exposure. Plot shows chronotype meta-analysis variants and their effects (log odds ratios) on major depressive disorder in the PGC GWAS[1] (outcome) versus odds of being a morning person (exposure). Lines identify the slopes of the five methods tested. Log odds (and SEs) for morningness were taken from the secondary effect-size meta-analysis. Error bars represent standard errors of effect sizes.

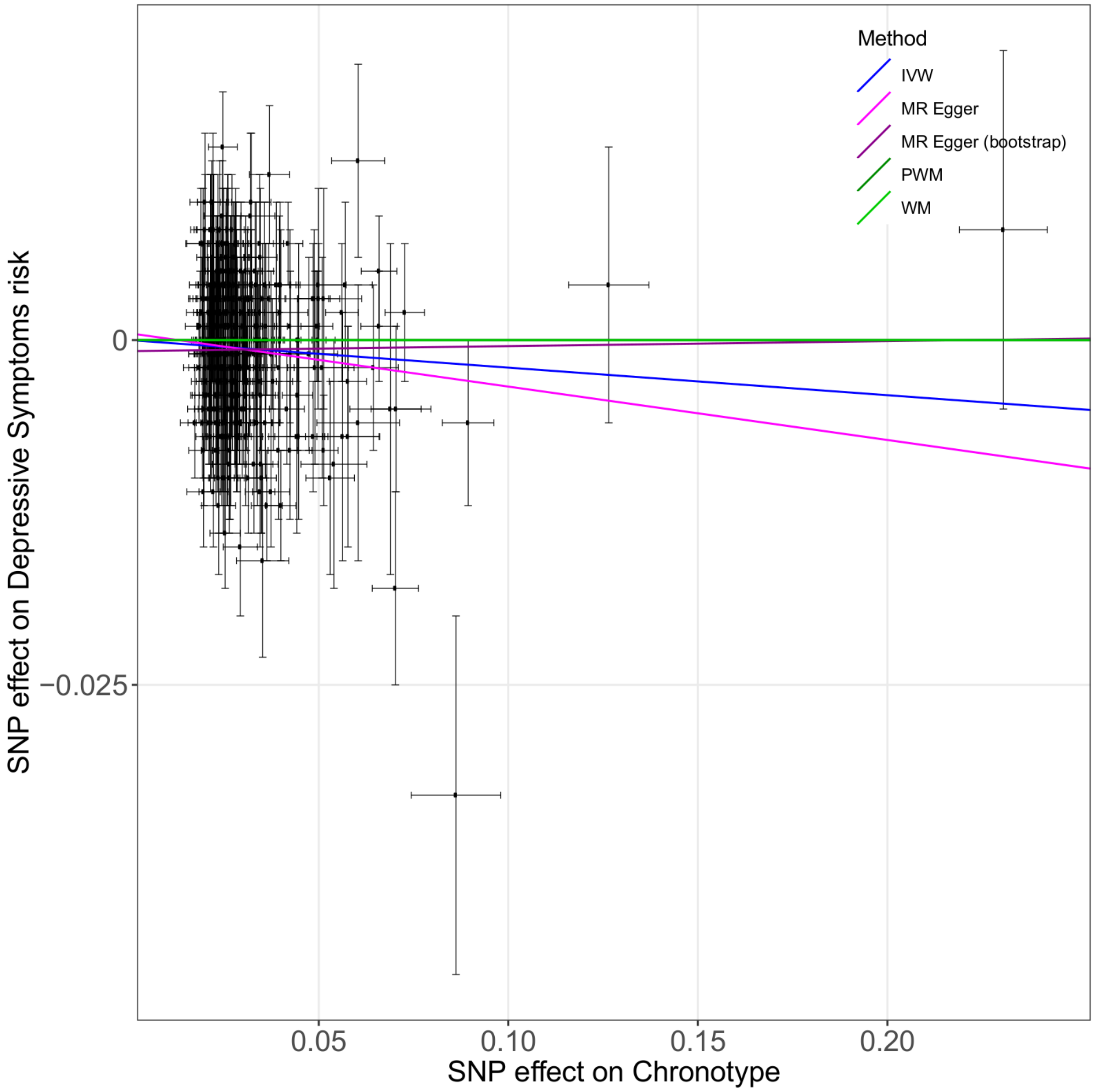

Supplementary Figure 4. MR scatter plot of risk of depressive symptoms vs. chronotype exposure. Plot shows chronotype meta-analysis variants and their effects (log odds ratios) on depressive symptoms in the SSGAC GWAS[2] (outcome) versus odds of being a morning person (exposure). Lines identify the slopes of the five methods tested. Log odds (and SEs) for morningness were taken from the secondary effect-size meta-analysis. Error bars represent standard errors of effect sizes.

Supplementary References

1. Sullivan, P. F. *et al.* A mega-Analysis of genome-wide association studies for major depressive disorder. *Mol. Psychiatry* **18,** 497–511 (2013).

2. Okbay, A. *et al.* Genetic variants associated with subjective well-being, depressive symptoms, and neuroticism identified through genome-wide analyses. *Nat. Genet.* **48,** 624–633 (2016).
