# Supplementary material for: Genome-wide association analyses of chronotype in 697,828 individuals provides insights into circadian rhythms
Source: Nat Commun. 2019 Jan 29;10:343. doi: 10.1038/s41467-018-08259-7 (PMC6351539; doi:10.1038/s41467-018-08259-7)
Supplement: Supplementary file 3 — Description of Additional Supplementary Files [file 41467_2018_8259_MOESM3_ESM.pdf]

Description of Additional Supplementary Files

Supplementary Data 1. Lead variants from the METAL meta-analysis of UK Biobank Chronotype and 23andMe morning person GWAS. As this was a P-value based meta-analysis, we provide the morningness ln OR from the secondary UK Biobank morning person and 23andMe morning person meta-analysis. Effect sizes and directions correspond to Allele1.

Supplementary Data 2. Loci with multiple independent signals and their respective lead variants, identified using GCTA-COJO. Lead variants identified only distance-based clumping are highlighted in green, whereas additional independent signals are highlighted blue. Betas and SEs were derived from the intercept-corrected Z-score from the meta-analysis as detailed in the Supplementary Note of Zhu et al, 2016; doi:10.1038/ng.3538.

Supplementary Data 3. GWAS summary statistics of the Chronotype lead variants for eight objective accelerometry-derived traits in the UK Biobank. Beta is reported for Allele1 and units are in hours, except for number of sleep periods (N) and sleep efficiency (%).

Supplementary Data 4. Gene-set enrichment results from FUMA (MAGMA) on 10894 gene sets. Bonferroni correction assumes 10,894 independent tests. The 37 pathways with P Bonf < 0.05 are highlighted in green.

Supplementary Data 5. PASCAL gene-set enrichment results ordered by empirical P.

Supplementary Data 6. Gene set enrichment results from MAGENTA. We used 110Kb upstream and 40Kb downstream limits for assigning variants to genes and with the number of permutations set to 10,000.

Supplementary Data 7. DEPICT gene set enrichment results.

Supplementary Data 8. DEPICT tissue enrichment results.

Supplementary Data 9. Finemapped chronotype loci with "plausible" variants (log10bf > 2) and their annotations. Alamut annotations were only considered for the longest transcript. GTEx annotations identify significant associations between variants and the expression of one or more genes in at least one brain tissue or at least one of any other tissue type. Genes identified by the GTEx eQTL associations, as well as nearby genes, were investigated for evidence of enrichment in the Suprachiasmatic Nucleus (SCN) and evidence of circadian fluctuation in expression levels (Pembroke et al., 2015, eLife, https://doi.org/10.7554/eLife.10518 and Brown et al., 2017, Nucleic Acids Res, https://dx.doi.org/10.1093/nar/gkx714). These same genes were cross-referenced against a database of genes identified as modifying the circadian clock through RNAi knockdown screening (Zhang et al., 2009, https://doi.org/10.1016/j.cell.2009.08.031).

Supplementary Data 10. Top 10,000 variants from the METAL meta-analysis of UK Biobank Chronotype and 23andMe morning person GWAS. We provide the morningness ln OR from the secondary UK Biobank morning person and 23andMe morning person meta-analysis as well as the uncorrected (non-LD score intercept corrected) Z-scores and P-values. Effect sizes and directions correspond to Allele1. Only 88 loci are represented in this table, as the top 10,000 variants are a subset of the 33,785 variants that reached genome-wide significance in the full meta-analysis.

Supplementary Data 11. Genetic correlations of Chronotype with 222 published phenotypes generated using LD Score Regression. P-values below the bonferroni-corrected threshold of 2.3E-4 are highlighed in green.

Supplementary Data 12. Results of two-sample MR analyses for Chronotype exposure against multiple outcomes. We used the TwoSampleMR package to test the 351 lead Chronotype variants against published GWAS outcomes, using the full range of tests. Results are sorted by inverse-variance weighted (IVW) P-values. Chronotype effect sizes were log odds ratios of morningness, taken from the secondary effect-size meta-analysis of UK Biobank morning person and 23andMe morning person GWAS.

Supplementary Data 13. Two-sample MR results for multiple exposures against Chronotype outcome. We used published variants and their effect sizes as instruments against Chronotype as an outcome. For all exposures except "Major depressive disorder (23andMe)", Chronotype effect sizes (log odds ratios of morningness) were taken from the secondary effect-size meta-analysis of UK Biobank morning person and 23andMe morning person GWAS. For the Major depressive disorder (23andMe) exposure, we used variants identified in https://doi.org/10.1038/ng.3623 (PMID 27479909) and outcome statistics from the UK Biobank chronotype GWAS.

Supplementary Data 14. R wrapper script used to call GGIR to process the converted (.wav) UK Biobank activity monitor files. All settings, except file locations, are identical to those used in this study.

Supplementary Data 15. List of lead variants at P<5E-8 identified in the 23andMe morning person GWAS alone. Some variants were not present in the UK Biobank (orange) and, where possible, alternative variants in the same locus that were present in the UK Biobank HRC-imputed panel (green) are listed. Some loci had no suitable alternative variants reaching P<5E-8 in the 23andMe only GWAS. Effect sizes (ln ORs) correspond to A1.
